# Impact of Duration of Macula off Rhegmatogenous Retinal Detachment on Visual Outcome

**DOI:** 10.12669/pjms.303.4744

**Published:** 2014

**Authors:** Mahtab Alam Khanzada, Shahid Wahab, Lakhani Das Hargun

**Affiliations:** 1 Dr. Mahtab Alam Khanzada MBBS, FCPS, Second Fellowship trainee (Vireo retina), Ophthalmology Unit I, Dow University of Health Sciences, Civil Hospital, Karachi, Pakistan.; 2 Dr. ShahidWahab MBBS, MCPS, FCPS, Professor of Ophthalmology, Ophthalmology Unit I, Dow University of Health Sciences, Civil Hospital, Karachi, Pakistan.; 3 Dr. Hargun Das Lakhani MBBS, FCPS, Assistant Professor, Ophthalmology Unit I, Dow University of Health Sciences, Civil Hospital, Karachi, Pakistan.

**Keywords:** Duration of Macula off, Scleral buckling, Visual outcome

## Abstract

***Objective:*** To assess impact of duration of macular detachment on visual outcome after scleral buckling for retinal detachment with macula off.

***Methods:*** Prospective, descriptive case series was conducted at Ophthalmology Department Dow University of Health Sciences, Civil Hospital Karachi and Al Noor Eye Clinic Karachi from May 2012 to June 2013. Five groups were made according to period of macular detachment. Best corrected Visual acuity (BCVA) was main outcome measure. P value < 0.001 was considered significant.

***Results:*** Mean duration of macula off was 17.0±4.0 (SD) days. Mean pre-operative VA in patients with immediate, early, intermediate, delayed or late group were 2/60, 2/60, Counting figure (CF) 3 meters (m), CF2 m and Hand Movement (HM) respectively. Only 48.48% patients **of** those repaired within 7 to15 days had significantly better (P < 0.001) BCVA (6/9-6/18) than the other groups. Only 19.35% patients of intermediate group achieved BCVA 6/18-6/24 (P < 0.001) which was comparatively better than the delayed and late group.

***Conclusions:*** Scleral buckle surgery for macular-off Rhegmatogenous Retinal Detachment has good post-operative visual outcomes if repaired within two weeks.

## INTRODUCTION

Rhegmatogenous Retinal Detachment (RRD) is the separation of neurosensory retina from retinal pigment epithelium (RPE) in association with accumulation of sub retinal fluid (SRF) in the presence of one or more retinal breaks. A significant amount of visual loss occurs especially when macula is involved.^1^^,^^2^ Longer duration of detached retina may lead to complete blindness^3^ due to permanent functional damage to the photoreceptors at the macula. However exact cut of period regarding how earlier fresh RRD with macula off should be repaired remain a debatable issue. Hassan and associated^4^, and Schwartz and coworkers^5^ agree that surgical repair of detached retina earlier than 10 days shows best results to restore visual acuity with macula off. Therefore retinal detachment (RD) should be considered urgency and must be repaired as early as possible particularly when macula is off.

The underlying principles in the scleral buckling (SB) are indentation of globe wall to provide RPE apposition to the neurosensory retina and closure of the hole / break by Cryotherapy. If the break is properly closed, the RPE pump actively absorbs sub retinal fluid from the sub retinal space and retina will attach spontaneously.^6^ Eventual visual out come after successful scleral buckling depends on some pre and post-operative factors such as age of patient, myopia, pre-operative visual acuity (VA), duration of macula off, height of macular detachment, extent of RD, number and location of retinal breaks, presence of PVR, post-operative cystoid macular edema and epiretinal membrane.^7^^,^^8^ Due to diverse presentation of RRD vireo retinal surgeons have to customize buckling procedure according to these factors individually or collectively.^7^^,^^8^

There is considerable work done in Pakistan for repair of RRD with scleral buckle and they focused on anatomical reattachment and visual out-come after surgery.^9^^,^^10^ In this study we are paying emphasis to determine the impact on visual out-come of the duration of macular detachment before surgery. This would hopefully guide vireo retinal surgeons to decide proper time of surgical intervention.

## METHODS

This prospective, descriptive study was carried out at the department of ophthalmology, Dow University of Health Sciences, Civil Hospital Karachi and at Al Noor Eye Clinic Karachi during the period of May 2012 to June 2013.

Convenience sampling technique was used for patient recruitment/data collection. One hundred and seventy adult phakic patients of either gender having primary Rhegmatogenous Retinal Detachment (RRD) with macula-off having VA of 6/60 or less and who were able to estimate the exact time period for onset of significant visual loss were selected from outpatient department. Those patients who have pseudophakic RD, unable to describe exact time of onset of visual loss, macula not involved, evaluation not possible, re-detachment with or without PVR, Retinoschisis, Giant Retinal Tear, Vitreous hemorrhage and prior or coexistent ocular disease/surgery were not included in our study. High myopic (> 6.0 Diopter) patients were not included because outcomes may vary while repairing retina in highly pathological myopia. Patients having diabetes mellitus or systemic hypertension were also excluded from this study.

All patients underwent a complete ophthalmic examination. Demographic and clinical data like sex, age, duration of RD, preoperative VA, intraocular pressure (IOP), relative afferent pupillary defect (RAPD) and lens status were recorded. The macular status, number and type of retinal breaks to choose the size and type of scleral buckles were determined with the help of triple mirror and indirect ophthalmoscope with indentation. Five groups were made in accordance with duration of macula off and labeled as immediate group (within a week), early group (within 2 weeks), intermediate group (within 4 weeks), delayed group (within 6 weeks) and late group (≥6 weeks).

 After counseling, informed written consent were obtained. All surgeries were done under general anesthesia. After peritomy rectus muscles were isolated and scleral incision was made to drain the SRF. Retinopexy was achieved by cryotherapy at the break site. Explants were placed according to type, size and location of the break. Segmental circumferential buckles were used in eyes with multiple retinal breaks which could not be covered by one or two radial buckles. Encircling buckling was performed only when limited scleral buckling was found to be inadequate and when there was total RD.

Postoperative topical corticosteroids with antibiotics were prescribed and tapered over the subsequent period till three weeks.The follow up was done on 1^st^ day, 1^st^ week, 2^nd^ week, one month and finally on three months. Postoperatively retinal attachment, macular status and visual out comes were recorded on each visit.

***Statistical Analysis:*** SPSS version 16.0 was used to analyze the data. Student’s t-test was used to compare mean values; qualitative variables were compared using the Chi-square test. P value < 0.001 was considered significant.

## RESULTS

Among the 170 patients, 95 males and 75 females with mean age of 51.50±15.6 (SD) years (ranged from 25 to 55 years) were enrolled. Right eye was operated in 60.58% cases while remaining 39.41% had left eye involvement. The range for duration of macula off was found from 5 to 55 days (mean 17.0±4.0 SD) (Table-I). The mean pre-operative VA in patients with immediate, early, intermediate, delayed and late groups were 2/60, 2/60, CF 3 meters, CF 2 m and HM + respectively. Post-operative BCVA was recorded in four categories; (1) 6/12 or better, (2) 6/18 to 6/24, (3) 6/36-6/60 and (4) less than 6/60 respectively. Post-operative visual out comes during follow up period is shown in Table-II. Twenty two patients were enrolled in immediate group and forty four patients were registered in early group. Statistically significant visual out comes (BCVA 6/9-6/12) was observed in 48.48% of those patients who were operated with in 1 to 15 days of their macula off (immediate & early groups). While in the intermediate group 19.35% of patients achieved BCVA (6/18-6/24) which was also found better as compared with delayed and late groups at the end of three months follow up (Table III). Two cases in each intermediate and delayed groups developed sub retinal bleeding per operatively at sub retinal fluid drainage site which resolved subsequently within two weeks.

**Table-I:** Demographic Data (n=170).

Gender	
Male	95 (55.88%)
Female	75 (44.11%)
Laterality	
Right Eye	103 (60.58%)
Left Eye	67 (39.41%)
Mean Age (years)	51.50 ±15.6 (SD)
Mean Duration of Macula Off (days)	17.0 ± 4.00 (SD)

**Table-II:** Comparison of pre and post-operative visual acuity (mean values).

*DMO* **	*BCVA 6/9-6/12* *% (n)*	*BCVA 6/18-6/24* *% (n)*	*BCVA 6/36-6/60* *% (n)*	*BCVA <6/60* *% ( n)*	*P Value*
Immediate ( n=22)	81.81(18)	18.18(04)	00.00(00)	00.00(00)	< 0.001*
Early ( n=44)	31.81(14)	63.63(28)	04.54(02)	00.00(00)	< 0.001*
Intermediate ( n=62)	00.00(00)	19.35(12)	61.29(38)	19.35(12)	< 0.021
Delayed ( n=22)	00.00(00)	13.63(03)	50.00(11)	36.36(08)	0.039
Late ( n=20)	00.00(00)	00.00(00)	10.00(02)	90.00(18)	0.499

* Statistically significant

** Duration of Macula off.

**Table- III:** Post-operative BCVA at the end of three months follow up

*DMO*	*Pre-op* *VA(n)*	*Post-op* *VA (n)* *1* ^st^ * Day*	*Post-op* *VA (n)* *1* ^st^ * week*	*Post-op* *BCVA (n)* *1* ^st^ * month*	*Post-op* *BCVA (n)* *3* ^rd^ * month*
Immediate	2/60(22)	6/24(22)	6/18(22)	6/12(18)	6/9(10)
Early	2/60(44)	6/36(44)	6/18(42)	6/12(10)	6/9(04)
Intermediate	CF 3m(62)	6/60(50)	6/36(31)	6/24(12)	6/18(07)
Delayed	CF 2m(22)	5/60(22)	6/36(11)	6/24(01)	6/18(02)
Late	HM+(20)	HM+(20)	5/60(09)	5/60(09)	6/36(02)

**Fig.1:**
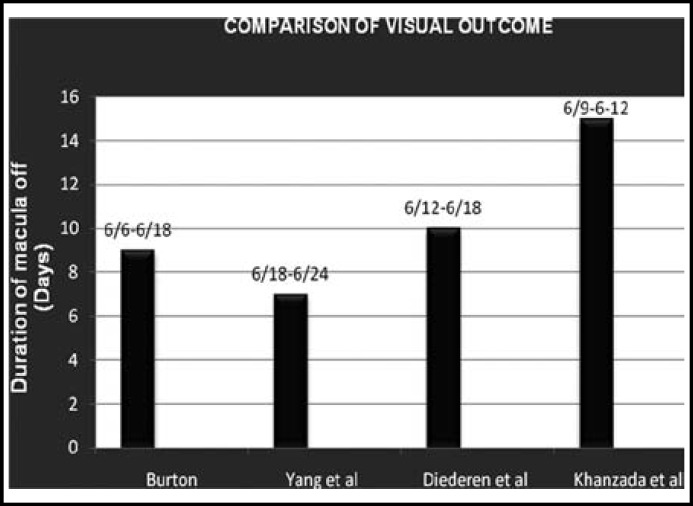
Comparison of visual outcome

## DISCUSSION

Scleral buckling procedure is a time tested standard for repairing RRD without proliferative vitreoretinopathy (PVR).^11^^,^^12^ It has initial 82 to 92% anatomic success rate^5^^,^^13^ in both phakic and pseudophakic patients with lesser number of complications.^14^^,^^15^ Although in the past decade pneumatic retinopexy and primary pars plana vitrectomy (PPV) with or without scleral buckle had set effective trends to repair RRD but scleral buckling alone seems to surpass vitrectomy in the treatment of phakic RRD.^16^^-^^20^

Exact cut off period for duration of macula off and surgical intervention is difficult to establish but majority agree that visual recovery is inversely proportional to the duration of macula off. According to Ross^7^, the duration of macular detachment within the first week does not influence the visual recovery after the RD surgery. Liu et al.^21^ also concluded that the SB surgery has no statistically significant impact on the final visual recovery in patients with an acute primary macular-off RRD of less than or equal to 7 days. Our study endorses the work of these authors (Fig.1). In our study, mean postoperative BCVA in immediate & early groups (1-2 weeks) were significantly better (p-< 0.001) compared to the intermediate, delayed and late groups (Table-III). Earlier Burton et al.^22^ achieved VA 6/6 to 6/18 in 53% patients with macula off RD who underwent surgery within nine days. In our study we found visual recovery 6/9 to 6/12 only in 48.48% of patients who reported in first two weeks compared to the late comers. Our results are also comparable with that of Yang^23^ and Diederen et al.^24^ (Fig.1).

Our study shows better visual out come on each succeeding follow up day than the previous visit (Table-II) due to further recovery of the photo pigments after the reattachment of retina. Long term visual impairment in post-operative period was found more in patients of younger age, no or mild myopia and shorter duration of macular detachment. Our findings support the study of Liem^25^ and Kusaka et al.^26^

Some studies^9^^,^^10^^,^^27^ concluded that status of macula at the time of surgery and level of proliferative vitreoretinopathy (PVR) influence the anatomical and functional out come after SB procedure. Duration of macula off does matter final visual recovery after unevent full buckling but height of macular detachment has very important role for predicting final visual outcome. Hagimura^28^ and Lecleire et al^29^ used OCT in macula off RRD and observed less disruption of the neurosensory retina with minimal elevation of retina at the macula. BCVA was more impaired in highly detached retina. This suggests that irreversible nutritional damage occurs to the macula in highly elevated detachment of short duration. These observations are good explanationas to why some macular detachments of short duration (even 1-2 days only) don’t gain better than 6/60 vision after early successful buckling surgeries. The limitation of our study is that we couldn’t use OCT and only relied on our clinical observation for height of macular elevation. Reasons other than duration of macula off not included in the objectives of the study.

There are some reported complications related to scleral buckle surgery such as buckle infection, diplopia, ocular motility disturbance, strabismus, myopia, external extrusion, trans-scleral (internal) erosion and potential retinal and choroidal ischemia.^4^ None of such complications were observed in our study during whole follow up period. In our study four cases developed sub retinal bleeding which resolved subsequently. This bleeding was away from the macula so it did not affect the visual outcome.

## CONCLUSION

The good visual outcome depends upon reattachment of macula as **earliest** possible i.e. within first two weeks otherwise further damage may compromise the final visual acuity. Longer the duration of macula off poorer will be visual outcome. 

## RECOMMENDATIONS

Many aspects of retinal detachment surgery are similar in developing and industrialized countries.^30^ But the result of surgical intervention differs due to lack of both primary eye care and specialist retinal centers for early intervention. So we need to develop skilled persons and specialist centers in our country where patients of retinal detachment can report early to get better visual outcomes. 
